# The Morphogenesis of Sperm Storage Micropockets in the Western Mosquitofish (*Gambusia affinis*)

**DOI:** 10.3390/ani15050707

**Published:** 2025-02-28

**Authors:** Tengfei Wu, Ping Li, Zechen Wu, Yongming Wang, Sheng Li, Feng Shao, Zuogang Peng

**Affiliations:** 1 Key Laboratory of Freshwater Fish Reproduction and Development (Ministry of Education), School of Life Sciences, Southwest University, No. 2 Tiansheng Road, Chongqing 400715, China; feiyuhanqing@outlook.com (T.W.); wuzechen0807@163.com (Z.W.); wym8188@126.com (Y.W.); risenl@163.com (S.L.); 2 College of Fisheries, Southwest University, Chongqing 402460, China; lp2006@swu.edu.cn

**Keywords:** western mosquitofish, Poeciliidae, sperm storage micropocket, protrusion, previtellogenic oocyte

## Abstract

Sperm storage within the female reproductive tract is widespread among animal species with internal fertilization, such as the fish family Poeciliidae, wherein the females possess a sperm storage micropocket (SSP) that can store sperm for up to a year. However, the underlying mechanism and morphogenesis of SSP remain unclear. Here, using histological analysis, we found that the SSP of the western mosquitofish (*Gambusia affinis*) was associated with protrusions arising from the previtellogenic oocyte (PO). Similar protrusions occur in the PO of *Poecilia reticulata*, *P. latipinna*, *Xiphophorus helleri*, and *X. maculatus*, suggesting that such PO-derived protrusions might be a universal feature in Poeciliidae. These protrusions exhibit significant differences in structural composition compared to the cytoplasm. Furthermore, the area of these protrusions positively correlates with the total area of the oocytes. After maturing, the protrusions split from the oocytes, leaving behind sac-like structures composed of germinal epithelium, which later develop into the SSP for storing sperm. The bottom of the SSP is composed of a single layer of germinal epithelium. We describe and depict the protrusion formation process in the fish family Poeciliidae during the previtellogenic stage, elucidating the SSP morphogenesis.

## Simple Summary

The females of species undergoing internal fertilization often possess specialized structures or organs in their reproductive system to store spermatozoa. Here, we investigated the formation of sperm storage structures in the western mosquitofish (*Gambusia affinis*), and we describe how a sperm storage micropocket (SSP) begins. This process is initiated when a protrusion forms on a previtellogenic oocyte, a feature that exhibits consistency in other poeciliid fishes. This protrusion grows, pushes through the outer layer of tissue, and breaks off into the ovarian lumen (we have also observed the emergence of similar protrusion structures in other poeciliid fishes). The sac-like space formed by the compression of the germinal epithelium due to the protrusion represents the initial formation of the SSP, after which a single layer of germinal epithelium forms at the base, developing into a mature SSP. This study reveals the reason for the formation of the SSP and its morphogenesis.

## 1. Introduction

Animals that undergo internal fertilization commonly demonstrate long-term sperm storage capability [1]. This ability allows these animals to adapt to diverse ecological environments by temporally separating mating from fertilization [2]. Furthermore, it significantly influences their life histories, mating systems, cryptic female choice, sperm competition, and sexual conflict [3,4]. In species with internal fertilization, the ability to store sperm varies across species. Among vertebrates, the ovaries of fish can preserve fertile sperm for over 1–2 years [1], whereas in reptiles, turtles have a longer period of effective sperm storage (up to 4 years) [5]. In birds, the longest recorded storage period is 117 days [1]. However, with the exception of bats, which can store sperm for several months, the majority of mammals exhibit a relatively short storage time, ranging from a few hours to a few days [1,3]. In invertebrates, the duration of sperm storage can be as long as 10 years (*Atta colombica*) [6] or as short as two weeks (*Drosophila melanogaster*) [7].

Most internally fertilized species have evolved specialized structures or organs for sperm storage. For example, the Trinidadian guppy (*Poecilia reticulata*) stores sperm in ovarian micropockets [8], the coastal tailed frog (*Ascaphus truei*) possesses oviductal sperm storage tubules [9], and the painted turtle (*Chrysemys picta*) stores sperm within the albumin-secreting glands located in the oviducts [10]. In birds, after mating, spermatozoa enter the sperm storage tubules located at the uterovaginal junction of the reproductive tract [1]. In dogs (*Canis lupus familiaris*), sperm has been observed to be mainly stored in the uterine glands and uterotubal junction after artificial insemination [11]. Ancestrally, dipterans possessed three spermathecae (long-term sperm storage organs), but *Drosophila* species retain only two of these structures [12].

Approximately 2% of known bony fish are viviparous [13]. Ovoviviparity in fish is complex and involves several evolutionary adaptations of the reproductive system, from oviparity to ovoviviparity, which requires internal fertilization [14]. Sperm storage is one of the most basic characteristics of the female reproductive system adapting to internal fertilization [2]. Sperm storage has been documented in Anablepidae [15], Cottidae [16], Embiotocidae [17], Sebastidae [16], and Poeciliidae [18]. Poeciliidae species are typically viviparous (except *Tomeurus gracilis*) and rely on internal (intrafollicular) fertilization. Furthermore, females of many species in this family have specialized reproductive tract structures for long-term sperm storage [19,20]. For example, in *Heterandria formosa*, a temporary sperm storage structure called a “solid plug” is formed by the closure of ovarian folds containing spermatozoa [18]. Kobayashi and Iwamatsu discovered a sperm storage structure in guppies, known as the sperm storage micropocket (SSP), and described its fine structure and dynamic development process [21]. López-Sepulcre found that the SSP in guppies could store viable sperm for up to 300 days [8]. In *H. formosa*, stored spermatozoa reside within the ovarian lumen, adjacent to maturing oocytes, positioned within “*delle*” (tubular structures that evolve from invaginations of the germinal epithelium bordering the ovarian lumen) [22]. In *Xiphophorus maculatus*, sperm is stored in deep surface pits and pockets [23]. The SSP is a structure commonly found in viviparous poeciliid fishes with intrafollicular fertilization. Although the sperm storage capacity of the Poeciliidae has been well studied [19], details around SSP formation in poeciliids remain unknown.

The western mosquitofish (*Gambusia affinis*) is well known for its global biological invasion, causing severe damage to local ecosystems and economies [24]. As a viviparous teleost, it is easy to breed, has strong stress resistance, and possesses a publicly available, high-quality genome, making it an essential research model for studying invasion biology [25]. The ovaries of female *G. affinis* can store sperm and maintain sperm viability for up to several months, even during non-breeding seasons, thereby allowing multiple broods of fry to be produced from a single mating [26,27]. In this study, using histological methods, we found that previtellogenic oocytes (POs) in *G. affinis* produce a cytoplasmic protrusion during both non-gestation and gestation. As the oocyte matures, this protrusion ruptures the germinal epithelium, flows into the ovarian lumen, and leaves behind a tubular structure formed by the invagination of the germinal epithelium, which is referred to as the SSP. Unlike sperm located on the surface of the ovarian lumen, those residing at the bottom of the SSP can establish direct contact with the oocyte, potentially enhancing fertilization success. We hypothesize that the formation of the SSP in poeciliid fishes plays a critical role in facilitating intrafollicular fertilization by enabling direct contact between sperm and the oocyte. Before this study, no study in the literature had directly explored the reasons and morphogenesis behind the formation of the SSP in poeciliid fishes. This study describes SSP formation in *G. affinis* and offers insights into their sperm storage mechanism during intrafollicular fertilization.

## 2. Materials and Methods

### 2.1. Animals

In total, 20 juvenile females and 50 adult females *Gambusia affinis* were collected from Chongde Lake of Southwest University (29°82′ N, 106°42′ W), China. *Poecilia reticulata*, *P. latipinna*, *Xiphophorus helleri*, and *X. maculatus* (3 females of each species) were purchased from Mingyi Aquarium in Beibei Flower Market in China (29°81′ N, 106°41′ W). Specimens were identified by examining their morphological characteristics. In our laboratory, fish were maintained in freshwater at 26 °C under a 14:10 h light–dark photoperiod cycle in a closed water recirculation rearing system. This protocol was conducted according to the guidelines for the ethical review of experimental animal welfare at Southwest University (accession number: SWU_LAC2024030066).

### 2.2. Histological Observation and Analysis

The collected fish were sedated using MS-222 (Macklin, Shanghai, China) at a concentration of 200 mg/L for 2 min and subsequently euthanized by pithing using a sharp instrument. The abdominal cavity was opened by a lateral incision, and the ovaries were quickly excised and fixed in 4% paraformaldehyde (Biosharp, Hefei, China) for 24 h. After fixation, the ovaries were washed in water, dehydrated in a series of graded alcohol (50%, 70%, 80%, 95%, 100%), cleared in xylene, and embedded in paraffin at 60 °C. The embedded ovaries were serially sectioned at 5 μm thickness in a longitudinal plane using a rotary slicer (Leica RM2235, Wetzlar, Germany) and stained with hematoxylin–eosin (H&E) and Masson’s trichrome and periodic acid–Schiff (PAS) staining kits (Solarbio, Beijing, China). For continuous sections, we targeted objective fields of view that had large protrusion areas and oocyte areas. Digital photomicrographs were obtained using a BX53 microscope (Olympus, Tokyo, Japan), which was equipped with interchangeable ×4, ×10, ×20, ×40, and ×100 objective lenses, and during the imaging process, primarily the ×4, ×40, and ×100 objectives were utilized. The microscope was also coupled with a camera adapter (model U-TV0.5XC-3, Olympus, Tokyo, Japan). The processing of photomicrographs, including background removal, brightness, and contrast balancing, and the addition of image labels, was performed using Adobe Photoshop 2024 and Adobe Illustrator 2024 software.

We used ImageJ (version 1.54d; National Institutes of Health, USA, https://imagej.org) [28] software to measure the area and gray values of protrusions and oocytes, which had not previously been investigated in this domain. Gray values (0–255) quantify differences in histological sections [29], aiding in analyzing H&E staining variations between protrusions and oocytes. Each visual field was measured at least thrice, and the average of these measurements was calculated. The area of the protrusions was compared with the area of the corresponding oocytes to determine their size relationship. Similarly, the gray values of the protrusions and their corresponding cytoplasm were used to indicate the differences in staining intensity between them. We measured the total area of the protrusions and oocytes in 69 visual fields and assessed the gray values of the protrusions and cytoplasm in 67 visual fields. In area-related analysis, we conducted normality tests, performed Pearson correlation analysis, and applied a two-tailed paired t-test to assess significance. When analyzing gray values between the protrusions and cytoplasm, we utilized a two-tailed paired t-test to evaluate data differences. The α value (significance level) was set at 0.05. All analyses were performed using GraphPad Prism 8.2.1.

## 3. Results

### 3.1. Morphology of Protrusion During Previtellogenesis

**Figure 1:**
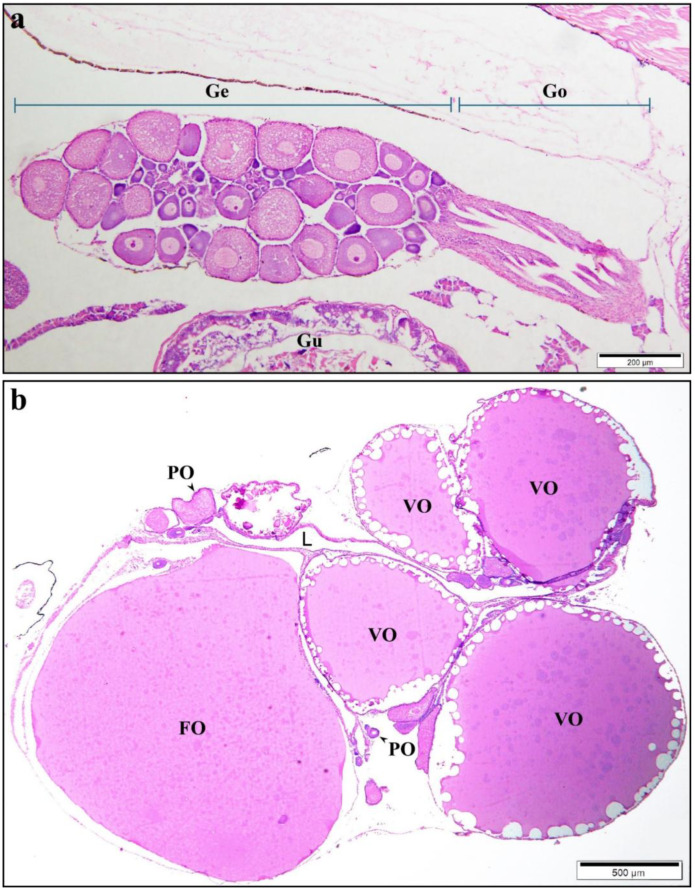
Panoramic view of the *Gambusia affinis* ovary showing previtellogenic oocytes, vitellogenic oocytes, and full-grown oocytes. (**a**) The longitudinal section of the juvenile ovary, showing two portions, the germinal zone and the gonoduct. The reproductive area contains previtellogenic oocytes and vitellogenic oocytes. (**b**) The longitudinal section of the adult ovary showing previtellogenic oocytes, vitellogenic oocytes, and fully grown oocytes. (**a**,**b**) H–E staining. Germinal portion (Ge), gonoduct (Go), gut (Gu), previtellogenic oocyte (PO), full-grown oocyte (FO), vitellogenic oocyte (VO), ovarian lumen (L).

In western mosquitofish juveniles, the ovary is a solitary saccular organ with a central lumen, located dorsal to the digestive duct and suspended from the dorsal wall of the body by the mesovarium (Figure 1a). The folds of the ovarian wall and lamellae project into the lumen, and numerous spermatozoa are stored in these folds during gestation. These folded structures that store sperm are called SSPs. All poeciliid fishes, which lack oviducts, have two zones: the germinal part and the gonoduct (Figure 1a). The gonoduct acts as a barrier between the ovarian germinal zone and the external environment. The germinal zone in adults contains oocytes that are in asynchronous development, including POs, vitellogenic oocytes (VOs), and full-grown oocytes (FOs) (Figure 1b). Sperm moves to the SSPs in the germinal zone via the gonoduct. Our current study has found that SSPs may be created by protrusions formed by POs. In *G. affinis*, the protrusion formed by the PO stains deeper red with eosin than the cytoplasm does. The protrusion squeezes the adjacent germinal epithelium to the side, leaving no observable germinal epithelium on top of the protrusion. Similar structures were observed in other Poeciliidae fish sampled in this study (Supplementary Figure S1).

**Figure 2:**
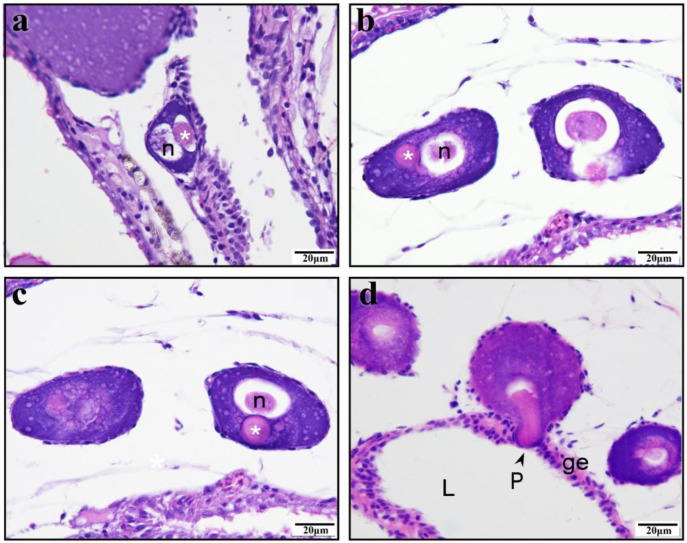
The protrusion in the pre-protrusion stage, when the oocyte is in the previtellogenic stage, in *Gambusia affinis*. (**a**) The pre-protrusion is stained purplish red (marked by a white asterisk), and chromatin is stained blue. (**b**,**c**). The pre-protrusion is stained purplish red (marked by a white asterisk), close to the edge of the oocyte. (**d**) The pre-protrusion stained purplish red by eosin and located at the edge of the oocyte, partitioning the continuous germinal epithelium. The top of the pre-protrusion has virtually no germinal epithelium and is exposed directly to the ovarian lumen. (**a**–**d**) H–E staining. Germinal epithelium (ge); ovarian lumen (L); protrusion (P); nucleus (n).

**Figure 3:**
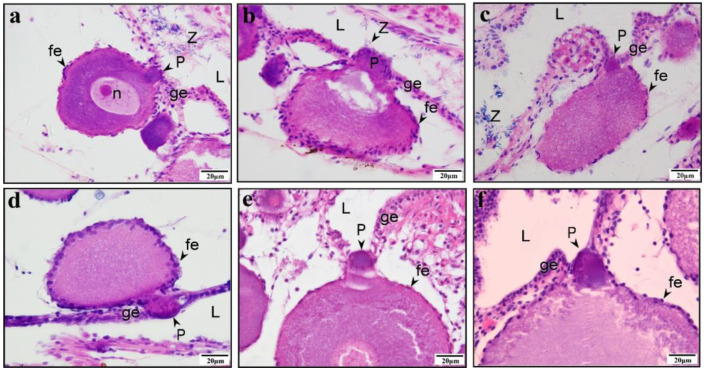
The protrusion at the growth stage and the oocyte at the previtellogenic stage in *Gambusia affinis*. (**a**) The protrusion is stained dark red by eosin and embedded in the germinal epithelium. (**b**) The protrusion pushes up the adjacent germinal epithelium, forming a bulge; many sperm are present in the ovarian lumen nearby. (**c**) The protrusion is stained dark red by eosin. A gap begins to appear in the continuous germinal epithelium. The germinal epithelium at the top of the protrusion is thinner, and many sperm are distributed in the ovarian lumen nearby. (**d**–**f**) The protrusion structure is deeply stained, distinct from the cytoplasm; no germinal epithelium is present on top of the protrusion. The protrusion separates the originally continuous germinal epithelium, with the divided portions located on either side of the protrusion. (**a**–**f**) H–E staining. Germinal epithelium (ge); ovarian lumen (L); protrusion (P); follicular epithelium (fe); nucleus (n); spermatozoa (Z).

**Figure 4:**
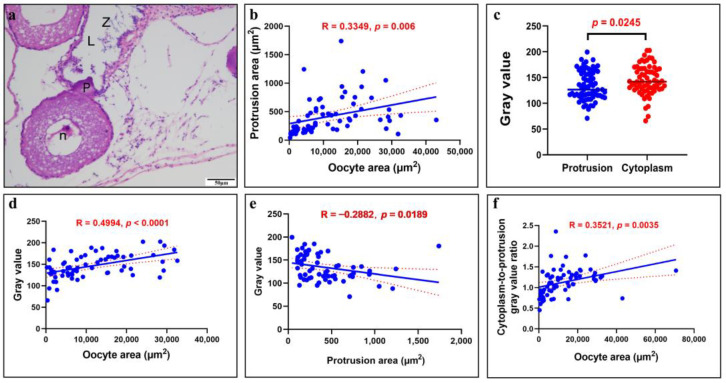
The area and gray value of protrusions and oocytes are significantly correlated. (**a**) Typical protrusion, with germinal epithelium on both sides, and the upper part of the protrusion located within the ovarian lumen. Spermatozoa (Z), ovarian lumen (L), protrusion (P), nucleus (n). (**b**) The protrusion area plotted against the oocyte area, with *n* = 66. The X-axis represents the oocyte area, and the Y-axis represents the corresponding protrusion area. Linear regression equation: Y = 0.01090 × X + 289.2. (**c**) A significant difference in the gray value between the protrusion and cytoplasm is seen (*n* = 67). The X-axis represents the protrusion and its paired cytoplasm, while the Y-axis represents the corresponding gray value. (**d**) The gray value plotted against the oocyte area (*n* = 65). The X-axis represents the oocyte area, and the Y-axis represents the corresponding gray value. Linear regression equation: Y = 0.001484 × X + 129.2. (**e**) The gray value plotted against the protrusion area (*n* = 66). The X-axis represents the protrusion area, and the Y-axis represents the corresponding gray value. Linear regression equation: Y = −0.02487 × X + 144.9. (**f**) Cytoplasm-to-protrusion gray value ratio plotted against the oocyte area (*n* = 67). The X-axis represents the oocyte area, and the Y-axis represents the corresponding cytoplasm-to-protrusion gray value ratio. Linear regression equation: Y = 9.412 × 10^−6^ × X + 1.009. The blue solid line represents the best-fit linear correlation (using Pearson’s product moment correlation coefficient via GraphPad Prism 8.2.1), and the red dashed line indicates the 95% confidence bands of the best-fit line. Significant correlation was assessed with Pearson’s product moment correlation coefficient using GraphPad Prism 8.2.1 (method = Pearson, two-sided, confidence interval = 95%). The α value (significance level) was set at 0.05.

The protrusion precursor in PO is visibly distinct from the cytoplasm and nucleus, appearing more brightly crimson under eosin staining and taking on a regular circular shape (Figure 2a–c). As the PO progresses to later stages, the protrusion precursor elongates, pressing towards the adjacent germinal epithelium (Figure 2d). During this development, the protrusion gradually grows into a structure that applies pressure on the epithelium, with numerous sperm concurrently observed in the nearby ovarian lumen (Figure 3a–c). The further growth of the protrusion results in significantly deeper red staining than that seen in the cytoplasm with eosin staining. The growing protrusion pushes the germinal epithelium towards both sides of the protrusion. The top of the protrusion remains devoid of germinal epithelium, gradually extending into the ovarian lumen (Figure 3d–f). The typical protrusion is distinctly delineated from the cytoplasm during staining (Figure 4a). We measured the areas of protrusions and oocytes in 66 paired visual fields (excluding 3 outliers) and found a positive correlation between the area of the protrusion and the area of the oocyte (Figure 4b). To distinguish the staining differences between the protrusions and cytoplasm, their gray values were measured. The measurement data from 67 protrusion and cytoplasm pairs indicated that the gray value of the cytoplasm was significantly higher than that of the protrusions (Figure 4c). Furthermore, the gray values of the cytoplasm and oocyte size were significantly positively correlated (Figure 4d), and the gray values of protrusions and protrusion size were significantly negatively correlated (Figure 4e). Meanwhile, a significant positive correlation was found for the cytoplasm-to-protrusion gray value ratio against the oocyte area (Figure 4f).

### 3.2. The Protrusion Flows into the Ovarian Lumen

**Figure 5:**
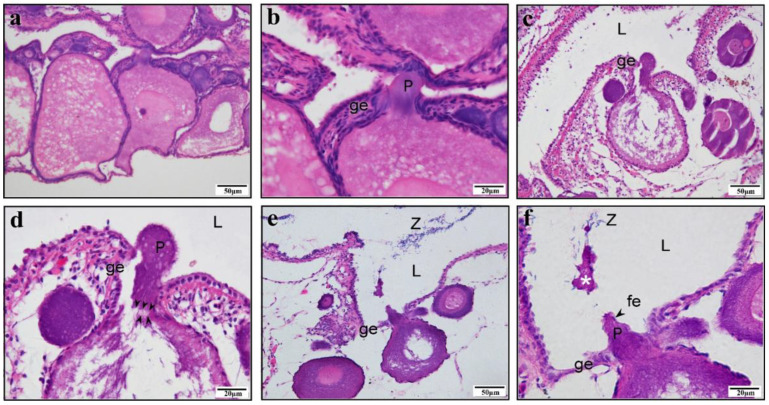
In *Gambusia affinis*, the mature protrusion breaks through the germinal epithelium, and its contents flow into the ovarian lumen. (**a**) Oocyte of a virgin fish before vitellogenesis. (**b**) Detail from image a. The protrusion and the germinal epithelium on both sides are deeply stained. The protrusion flows into the ovarian lumen. (**c**) Previtellogenic oocyte. The protrusion flows into the ovarian lumen. (**d**) Image c in detail: the protrusion is deeply stained, flowing into the ovarian lumen. A furrow begins to appear at the base of the protrusion (marked by a black arrow). The cytoplasm becomes vacuolated. (**e**) Previtellogenic oocyte with protrusion rupture. (**f**) Image e in detail: the upper part of the protrusion (marked by a white asterisk) is completely separated from the oocyte and flows into the ovarian lumen. Numerous sperm can be seen gathered around the contents of the protrusion. The edge of the base of the protrusion has loosely arranged follicle cells. The cytoplasm becomes vacuolated. (**a**–**f**) H–E staining. Germinal epithelium (ge); ovarian lumen (L); protrusion (P); follicular epithelium (fe); spermatozoa (Z).

As the PO of *G. affinis* continues to grow, the protrusion also becomes larger. The protrusion still stains deeper than the cytoplasm with eosin. It completely emerges from the germinal epithelium, which is located on the side of the protrusion, and flows into the ovarian lumen (Figure 5a, b). As the protrusion continues to flow into the ovarian lumen, a vacuole begins to form inside the oocyte (Figure 5c, d). The base of the protrusion begins to form a furrow, gradually detaching from the oocyte and flowing completely into the ovarian lumen. The edge of the base is bordered by sparse follicle cells, and many sperm are observed near the part that flows into the ovarian lumen (Figure 5e, f). We observed the same phenomenon of protrusions flowing into the ovarian lumen in the ovaries of other poeciliid fishes that we sampled as part of this study (Supplementary Figure S2).

### 3.3. Morphology of a Matured SSP

**Figure 6:**
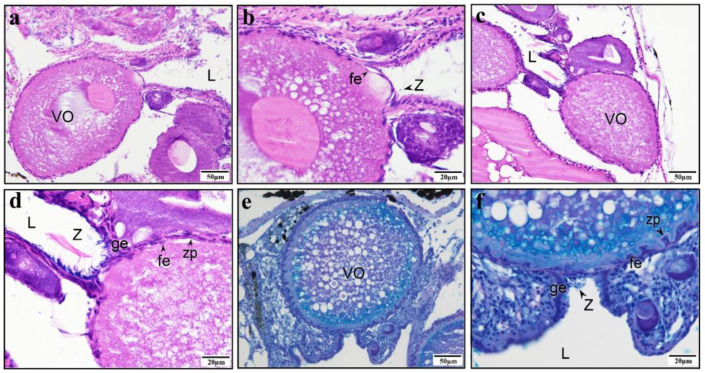
Sperm storage micropocket (SSP) formation in *Gambusia affinis*. (**a**) During vitellogenesis, the protrusion of the oocyte shrinks, and the oocyte is recovered. (**b**) Detail of image a. The protrusion is lined with germinal epithelium outside and follicle cells inside, with sparse sperm nearby. (**c**) The protrusion disappears during vitellogenesis. (**d**) Detail of image c. Numerous sperm aggregate in the SSP, with their tails coiled together and heads embedded around and at the base. (**e**) During vitellogenesis, the SSP is formed. (**f**) Detail of image e. Follicle cells are embedded in indentations of the zona pellucida, with a few sperm visible at the base of the SSP. The oocyte and zona pellucida are stained purple-red. (**a**–**e**) H–E staining. (**e**,**f**) PAS. Ovarian lumen (L), vitellogenic oocyte (VO), spermatozoa (Z), germinal epithelium (ge), follicular epithelium (fe), zonal pellucida (zp).

**Figure 7:**
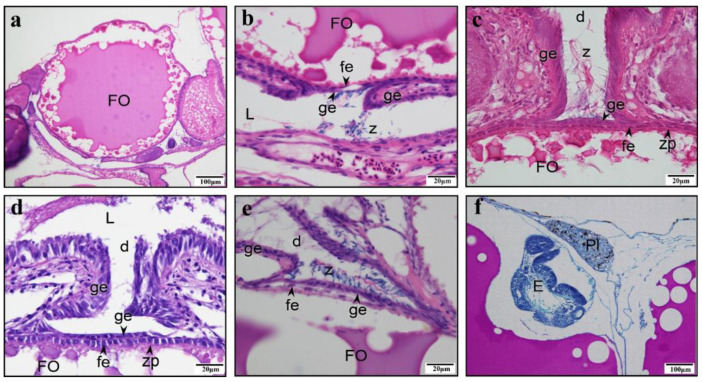
Developed sperm storage micropocket (SSP) in *Gambusia affinis*. (**a**) Full-grown oocyte with SSP. (**b**) Detail of image a showing that numerous sperm are gathered at the SSP and the single-layer follicular epithelium is adjacent to the single-layer germinal epithelium. Between the follicular epithelium and the germinal epithelium, a gap can be observed. (**c**) A full-grown oocyte with a well-developed SSP. Numerous sperm have gathered in the SSP, their tails intricately entangled. The single-layer follicular epithelium lies adjacent to the single-layer germinal epithelium. (**d**) A full-grown oocyte with a well-developed SSP. The bottom of the SSP consists of a single layer of germinal epithelium, but it is discontinuous, with gaps observed at the bottom edge. (**e**) A full-grown oocyte with a well-developed SSP. Numerous sperm can be observed at the bottom of the SSP. The single layer of germinal epithelium cells at the bottom of the SSP are arranged closely, and the single-layer follicular epithelium cells are loose and few in number. (**f**) The embryo develops near the edge of the oocyte adjacent to the SSP. At this stage, the SSP is occupied by abundant connective tissue. (**a**–**e**) H–E staining. (f) PAS. Ovarian lumen (L), full-grown oocyte (FO), *delle* (d), spermatozoa (Z), germinal epithelium (ge), zonal pellucida (zp), follicular epithelium (fe), fertilization plug (Pl), embryo (E).

**Figure 8:**
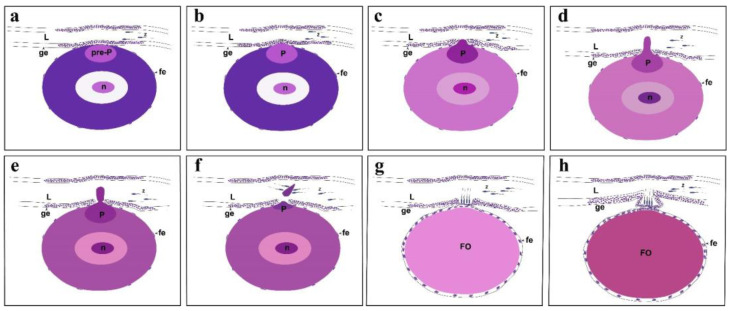
Conceptual summary. Figures show the forming of the protrusion and the morphogenesis of the sperm storage micropocket (SSP). (**a**) The pre-protrusion migrates to the edge of the oocyte, adjacent to the ovarian lumen. (**b**) As the protrusion forms, it begins to compress the ovarian lumen. (**c**,**d**) The protrusion penetrates through the germinal epithelium into the ovarian lumen and continues to grow. (**e**) A furrow begins to appear at the base of the mature protrusion. (**f**) The protrusion ruptures from the basal furrow and flows into the ovarian lumen. (**g**) The protrusion disappears, and the germinal epithelium forms a sac-like concave structure facing the oocyte. (**h**) The sac-like structure develops into a mature SSP, which can store spermatozoa. Ovarian lumen (L), pre-protrusion (pre-P), protrusion (P), nucleus (n), spermatozoa (Z), germinal epithelium (ge), follicular epithelium (fe), full-grown oocyte (FO).

When the protrusion flows into the ovarian lumen, oil droplets begin to appear in the oocyte (Figure 6a,b), which indicates that vitellogenesis has started. At this point, the base of the protrusion shrinks and the eosin stain becomes lighter (Figure 6a,b). A sparse layer of follicular cells is embedded at the edge of the protrusion, and there is a thin germinal epithelium outside, with a prominent gap (Figure 6a,b). After the protrusion completely disappears, the germinal epithelium forms a concave space facing the oocyte, which we refer to as the “*delle*”. In brief, the “*delle*” is a tubular structure formed by the invagination of the germinal epithelium, functioning as a sperm storage micropocket (SSP). After the complete disappearance of the protrusion, the germinal epithelium forms a concavity facing the oocyte, namely, the *delle*, which becomes the SSP when the sperm enters. The sperm heads are embedded at the bottom of the SSP and in the surrounding area, whereas the tails are regularly intertwined (Figure 6c,d). Currently, the oocyte is in the vitellogenesis stage. During the same period, follicular cells on the outer layer of the oocyte are embedded inward in the concavity formed by the zona pellucida, and a small amount of sperm can be observed at the bottom of the SSP (Figure 6e,f). As the oocyte continues to develop, the follicular cells on the outer layer of the oocyte are arranged regularly. The single layer of germinal epithelium at the bottom of the SSP does not adhere closely to the single layer of follicular cells, and obvious gaps can be observed. Many sperm are present at the bottom of the SSP and near the ovarian lumen (Figure 7a,b). The bottom of a more mature SSP comprises a single layer of germinal epithelium, adjacent to the single layer of follicular cells on the outermost layer of the oocyte. Numerous sperm heads are embedded at the bottom and surrounding areas of the SSP, with their tails intertwined (Figure 7c). The germinal epithelium at the bottom of the SSP is not continuously arranged, and there is a lack of germinal epithelium at the bottom edge (Figure 7d). When the oocyte matures, the follicular layer adjacent to the bottom of the SSP becomes loose and less numerous, and the bottom of the SSP also becomes thinner (Figure 7e). After the fertilization of the mature oocyte, the embryo develops near the edge of the SSP-adjacent oocyte, while the SSP is occupied by developing connective tissue (Figure 7f). The schematic diagram of the protrusion formation and SSP morphogenesis is presented in Figure 8.

## 4. Discussion

### 4.1. The SSP in Poeciliid Fishes

Sperm storage in the female reproductive tract is prevalent in species with internal fertilization, including mammals [30], reptiles [31], teleosts [32], insects [33], and birds [34]. Sperm-storing structures in the ovaries of poeciliid fishes have been reported in *Poecilia reticulata*, *Gambusia panuco*, *Xiphophorus hellerii*, *P. mexicana*, *P. formosa*, *Heterandria formosa*, etc. [19,21,22,32]. Fraser and Renton first demonstrated that *H. formosa* can temporarily store sperm in a structure formed by the closing of ovarian folds, which they termed a “solid plug” [18]. Kobayashi and Iwamatsu described a unique sperm retention structure within the ovary [21]. Later, Olivera-Tlahuel et al. used histological and stereological tools to identify and quantify the sperm storage structures in 12 species of poeciliid fishes, which they referred to as “spermathecae” [19]. The term “spermathecae” is more commonly used to describe specialized sperm storage structures in amphibians and insects [35,36]. In recent research, “SSP” has been adopted to describe the sperm storage structure in poeciliid fishes. We have a preliminary understanding of the phenomenon of sperm storage in the ovaries of poeciliid fishes and the structure of the SSP. However, there have been few reports on the causes and processes underlying SSP morphogenesis. Recently, we found that the PO of the western mosquitofish forms a protrusion that compresses the germinal epithelium during development. Subsequently, the base of the protrusion separates from the oocyte and flows into the ovarian lumen. We observed the same protrusion formation in the PO of other poeciliid fishes that we sampled as part of this study. This is the first study to report that poeciliid fishes exhibit protrusion formation in their POs. Once this protrusion disappears, the resulting sac-like structure formed by the compressed germinal epithelium serves as a storage site for sperm. Eventually, this structure evolves into an SSP.

### 4.2. Morphogenesis of the Protrusion

The ovary of the western mosquitofish is divided into a germinal part and a gonoduct. The gonoduct extends to the germinal part and differentiates into a narrower duct, namely, the ovarian lumen. The germinal part of the ovary contains follicles at different stages of development, including POs, VOs, and FOs. The ovarian lumen, located close to the oocyte, forms a micropocket for sperm storage. These characteristics are consistent with those of other poeciliid fishes [37,38].

During previtellogenesis, the conspicuous cytoplasmic structures observed under optical microscopy include oil droplets and cortical alveoli [39,40]. Oil droplets play a crucial role in the energy metabolism of eggs, serving not only as a reservoir of energy substrates but also as active intracellular structures that perform many other functions [41]. Cortical alveoli contain various glycoproteins that are released into the perivitelline space upon fertilization, effectively blocking polyspermy [42]. Recently, we discovered a unique structure in POs that is distinct from both oil droplets and cortical alveoli. This structure resembles a large oil droplet and gradually migrates towards the cell edge as the oocyte develops. The protruding structure may have participated in the formation process of the SSP. This stage, which we have termed “pre-protrusion,” has not been reported in the oocytes of poeciliid fishes. The pre-protrusion squeezes the edge of the oocyte to form a true protrusion, while pushing the adjacent germinal epithelium to both sides of the protrusion. This disrupts the continuous germinal epithelium. Interestingly, protrusions are observed in both virgin and gestating fish, suggesting that the formation of these protrusions does not rely on sperm induction. The protrusion appears transiently, emerging during the previtellogenesis stage and disappearing as it separates from the oocyte at the onset of vitellogenesis. To quantitatively describe the relationship between protrusions and oocytes, we measured the areas of both. The results demonstrated that during the previtellogenesis stage, the size of the protrusion and the size of the oocyte are significantly positively correlated, indicating that protrusion growth accompanies oocyte development. Furthermore, we observed sperm aggregation around the protrusions. The female reproductive tract interacts with sperm in various ways (both physical and molecular) to facilitate sperm migration towards the oocytes [43]. The contents of the protrusion appear to be related to the attraction of sperm to the oocytes.

The gray values can be used to quantify the pathology of H&E-stained rat lung sections [29]. In this study, we quantify the differences between protrusions and oocyte cytoplasm by measuring gray values, thereby reflecting their relationship. Various gray values indicate different compositions, implying that protrusions are special oocyte structures, not due to developmental irregularities. The gray values of the protrusions were significantly lower than those of the cytoplasm, suggesting distinct differences in their compositional makeup, such as variations in proteins and other cellular constituents. Furthermore, a significant negative correlation was observed between the gray values of the protrusions and their sizes, whereas a significant positive correlation was found between the gray values of the cytoplasm and the sizes of the oocytes, indicating that the compositional makeup of both changes with oocyte growth. The ratio of the gray value of the cytoplasm to that of the protrusions represented the degree of change in the compositional makeup, which showed a significant positive correlation with the size of the oocytes, suggesting a more pronounced change in the structural composition of the cytoplasm. Therefore, through a quantitative analysis of gray values, we found that the protrusions have a distinct structure that differs from the cytoplasm.

Notably, this phenomenon exists in both non-gestating and gestating fish, implying that the formation of protrusions does not depend on insemination or fertilization. Moreover, the present study has demonstrated that protrusions forming during the previtellogenic stage are also present in the ovaries of *Xiphophorus helleri*, *Poecilia reticulata*, *P. latipinna*, and *X. maculatus*. Protrusions formed during the previtellogenic stage may be ubiquitous in poeciliid fishes, making this phenomenon prevalent in these fishes.

### 4.3. The Protrusion Closely Resembles the First Polar Body

Various biological processes in the oocyte involve protrusion, including meiosis [44], sperm–egg binding [45], and interactions with adjacent somatic cells [46]. The formation and division of oocyte protrusions in the western mosquitofish is similar to the production of the first polar body. Mammalian oocytes undergo highly asymmetric division during meiosis, which produces a larger secondary oocyte and a very small first polar body [47]. During the formation of the first polar body, furrowing occurs at its base, resulting in the division of the secondary oocyte and polar body [44]. In the western mosquitofish, as the protrusion breaks through the germinal epithelium, it begins to enter the ovarian lumen. The protrusion continues to grow until most of it enters the ovarian lumen. At the base, a furrow begins to form that eventually splits the protrusion from the oocyte, allowing it to flow completely into the ovarian lumen. A few follicular epithelial cells are observed at the edge of the oocyte.

The nature of protrusions in the western mosquitofish remains unclear. However, the protrusion shares similarities with the first polar body in the following ways: (a) the protrusion forms before vitellogenesis, whereas the first polar body arises from the primary oocyte; (b) the protrusion is present in both inseminated and non-inseminated females; and (c) the protrusion size was significantly positively correlated with oocyte size, resembling the first polar body-oocyte size relationship reported in mice [44,47,48,49]. These characteristics indicate that the protrusion may be related to the morphogenesis of the first polar body. However, during the continued development of the oocyte, the fates of the protrusion and the first polar body diverge significantly. In many animals, the first polar body, once produced, remains within the zona pellucida until it degenerates and disappears [50]. In contrast, the protrusion in this study divides with the oocyte and is expelled into the ovarian lumen.

### 4.4. The Matured SSP

The SSP is a saccular structure formed by the protrusion generated by the PO pressing against the germinal epithelium. As the protrusion separates from the oocyte and the residual basal protrusion gradually diminishes, the oocyte recovers, leaving behind a saccular space. The bottom of this space, which is the outer side of the follicular epithelium, gradually forms a continuous single layer of germinal epithelium. Together with the saccular space, this layer constitutes a saccular structure, namely, the preliminarily formed SSP. The bottom of the SSP is adjacent to a single layer of follicular cells outside the oocyte; however, the two are not tightly attached, and obvious gaps can be observed. This loose attachment may be related to the subsequent disappearance of the single-layered germinal and follicular epithelia. In insects, including *Polistes jokahamae* and *Pachycondyla chinensis*, the spermatheca is a sac-like organ derived from the gonoduct, which stores sperm for a long time [51]. Interestingly, during the morphogenesis of the spermatheca in *Drosophila melanogaster* (approximately 48 h after puparium formation), a protrusion is also formed; however, unlike in this study, this protrusion is not formed by oocytes but rather by the sac-like structure itself [52]. This process of protrusion formation may facilitate the development of sac-like structures.

Many spermatozoa heads are embedded at the bottom of the SSP and the surrounding sac wall. The heads of spermatozoa are always embedded within germinal epithelial cells, indicating that these cells provide support for stored spermatozoa [53]. The tails of the sperm are entangled regularly, and this stable arrangement is conducive to the long-term storage of sperm. In the western mosquitofish, sperm are distributed at the bottom of the SSP or on the surface of the ovarian lumen. Similarly, in *X*. *maculatus*, two forms of association have been observed: sperm located within deep surface pits and pockets and spermatozoa taken up and integrated within the cytoplasm of specific epithelial cells [23]. When the oocyte matures, the follicle cells shed, and the germinal epithelium at the bottom of the SSP becomes sparse, with gaps observed. This phenomenon may be related to sperm breaking through obstacles in combination with oocytes. When mature oocytes are fertilized, the embryo develops at the edge of the oocyte close to the SSP (this location is the animal pole), and the gap in the spermatophore is replaced by abundant connective tissue, which is called the fertilization plug [22].

Moreover, other parts of the ovarian lumen can store sperm [23], but these sperm cannot directly contact the oocyte. Only the sperm stored in the SSP can directly contact mature oocytes. Why is it necessary to form a protrusion to create an SSP? We notice that the bottom of SSP is a single layer of germinal epithelium, which means that there are fewer obstacles to the achievement of intrafollicular fertilization. When the oocyte matures, the follicle cells shed, and at the same time, the bottom cells of the SSP disappear [22], allowing the sperm and oocyte to meet, achieving intrafollicular fertilization. Although the outflow of cytoplasm results in the loss of cytoplasm, this approach of forming an SSP at the bottom of a single layer of the germinal epithelium is crucial in intrafollicular fertilization.

Many pressing questions that need to be addressed still persist following this study, such as what exactly the protrusion is and the molecular mechanism behind its production. The formation of a single layer of germinal epithelium at the base of the SSP and its subsequent disappearance also require further investigation.

## 5. Conclusions

The presence of a structure for sperm storage in their female reproductive system is likely widespread among viviparous fishes. In this study, we have discovered that the structure in which poeciliid fishes store sperm originates from protrusions formed by previtellogenic oocytes. These protrusions exhibit significant differences in gray values compared to the cytoplasm, indicating that they are distinct structures compositionally separate from the cytoplasm. The area of these protrusions positively correlates with the total area of the oocytes, suggesting that they grow in conjunction with oocyte development. Subsequently, the protrusions split from the oocytes, leaving behind sac-like structures that later develop into SSPs for storing sperm. Additionally, the growth pattern of the protrusion resembles that of the first polar body, hinting at a possible connection between them. The protrusion forms the SSP with a single layer of germinal epithelium at the bottom by squeezing the germinal epithelium, which may be conducive in intrafollicular fertilization. Meanwhile, our observation of a similar phenomenon in other poeciliid fishes suggests that this could potentially be a conserved process within the family Poeciliidae.

## Data Availability

The article contains the relevant data and presents the original contributions of the study. For any further inquiries, please contact the corresponding author.

## Supplementary Materials

The following supporting information can be downloaded at: https://www.mdpi.com/article/10.3390/ani15050707/s1, Figure S1: Protrusions found in previtellogenic oocytes in Poeciliidae; Figure S2: The protrusion formed on the previtellogenic oocyte in Poeciliidae breaks through the germinal epithelium, releasing its contents into the ovarian lumen.
