# Supplementary material for: The Morphogenesis of Sperm Storage Micropockets in the Western Mosquitofish (Gambusia affinis)
Source: Animals (Basel). 2025 Feb 28;15(5):707. doi: 10.3390/ani15050707 (PMC11899314; doi:10.3390/ani15050707)
Supplement: Supplementary file 1 [file animals-15-00707-s001.zip › animals-3351268-supplementary.pdf]

Supplementary figures

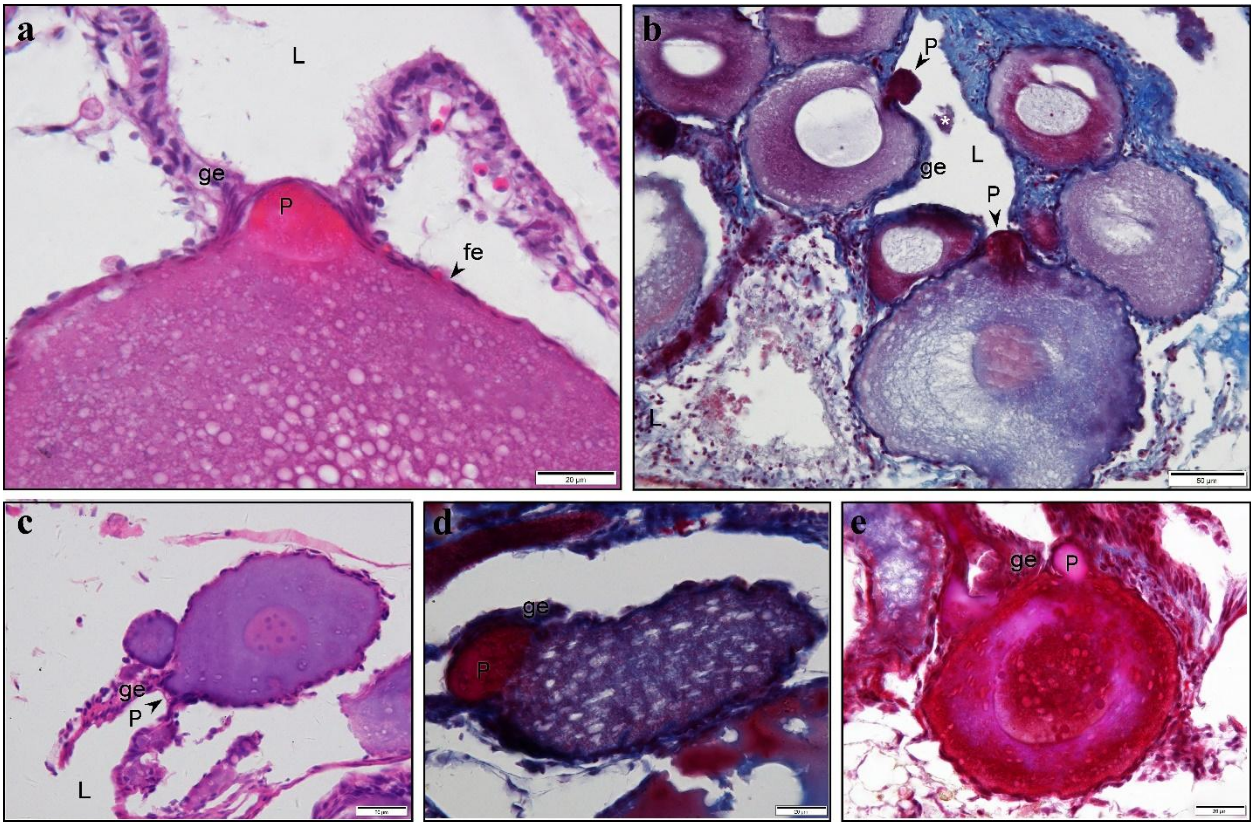

Figure S1. Protrusions found in previtellogenic oocytes in the Poeciliidae. (a) *Gambusia affinis;* the continuous germinal epithelium is on the side of the protrusion, whereas no germinal epithelium is present on the top of the protrusion. (b) *Xiphophorus helleri*; protrusions are stained purple-red, separating the continuous germinal epithelium and exposing them within the ovarian lumen. Scattered content of the protrusions (marked by white asterisks) can be observed nearby. (c) *Poecilia reticulata*; the protrusion stain is not markedly different from that of the cytoplasm, and the protrusion part separates the continuous germinal epithelium. (d) *Poecilia latipinna*; the protrusion, stained dark red, is almost completely within the ovarian lumen. (E) *Xiphophorus maculatus*; the protrusion formed by the oocyte is enclosed within the germinal epithelium, with a thinner layer of germinal epithelium at the top of the protrusion, which is adjacent to the ovarian lumen. a, c: H–E staining. b, d, e: Masson trichrome stain. Germinal epithelium (ge); ovarian lumen (L); protrusion (P); follicular epithelium (fe).

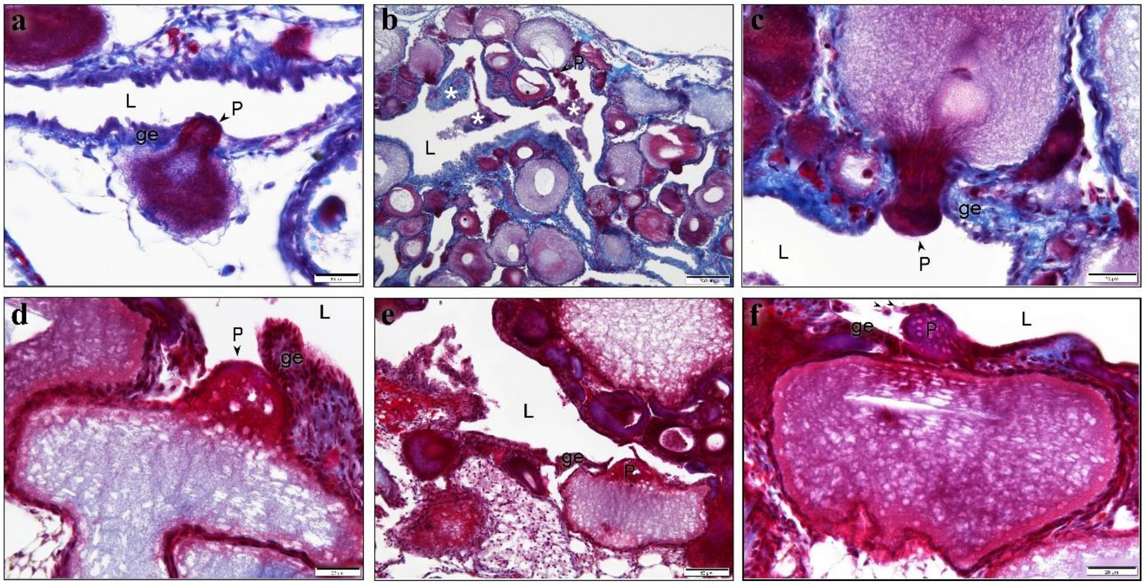

Figure S2. The protrusion formed on the previtellogenic oocyte in the Poeciliidae breaks through the germinal epithelium, releasing its contents into the ovarian lumen. (a) *Poecilia latipinna*; the protrusion pushes the adjacent germinal epithelium to one side, forming a bulge, and the germinal epithelium at the top of the protrusion is thinner. (b) *Xiphophorus helleri*; a large amount of protrusion content (white asterisk) inside the ovarian lumen is observed. (c) *X. helleri*; the protrusion is formed on the previtellogenic oocyte, stained deep red, without follicle cells on top, pushing aside the germinal epithelium and flowing into the ovarian lumen. (d) *Xiphophorus maculatus*; the protrusion is bright red, pushing the germinal epithelial layer aside. Meanwhile, the continuous germinal epithelium is broken, and the oocyte has an irregular form. (e) *X. maculatus*; the protrusion is stained bright red, and the adjacent germinal epithelium has been pushed aside and ruptured. (f) *X. maculatus*; scattered germinal epithelia (indicated by the black arrows) are seen at the top of the protrusion. a–f: Masson trichrome stain. Germinal epithelium (ge); ovarian lumen (L); protrusion (P).
